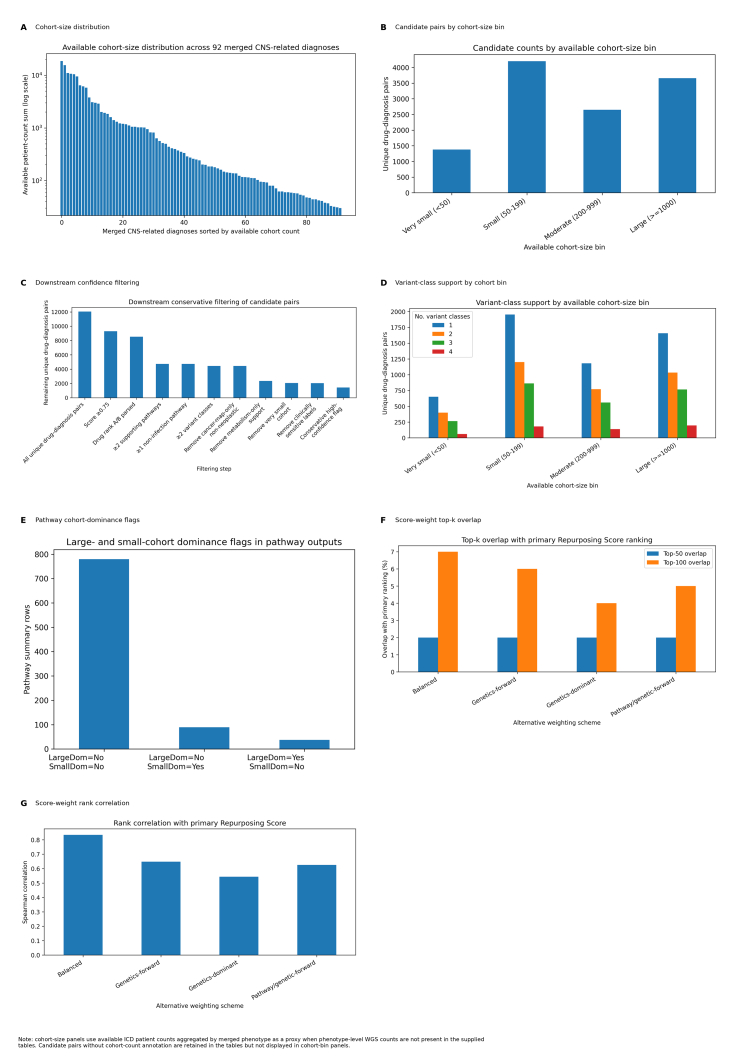# Drug repurposing opportunities across 92 CNS-related conditions using deep learning and whole-genome sequencing

**DOI:** 10.1016/j.neurot.2026.e00976

**Published:** 2026-07-28

**Authors:** Yichuan Liu, Hui-Qi Qu, Frank D. Mentch, Xiao Chang, Jihoon Kim, Haijun Qiu, Kayleigh Ostberg, Kenny Nguyen, Joseph Glessner, Hakon Hakonarson

**Affiliations:** a Center for Applied Genomics, Children's Hospital of Philadelphia, Philadelphia, PA, 19104, USA; b Department of Pediatrics, The Perelman School of Medicine, University of Pennsylvania, Philadelphia, PA, 19104, USA; c Division of Human Genetics, Children's Hospital of Philadelphia, Philadelphia, PA, 19104, USA; d Division of Pulmonary Medicine, Children's Hospital of Philadelphia, Philadelphia, PA, 19104, USA; e Faculty of Medicine, University of Iceland, Reykjavik, Iceland

**Keywords:** Drug repurposing, Whole-genome sequencing (WGS), Pediatric CNS disorders, Electronic health records (EHR)

## Abstract

CNS-related conditions span tumors, vascular, neurodevelopmental, and psychiatric disorders, yet therapeutic development for CNS disorders continues to face persistent delays and high attrition due to blood–brain barrier constraints, biological heterogeneity, and limited predictive models. Drug repurposing can shorten development timelines but requires scalable, mechanism-grounded prioritization. We curated 213 approved/clinical-stage drugs with KEGG pathway signatures and integrated them with whole-genome sequencing (WGS) pathway-importance profiles from 4392 individuals across 92 diagnoses. For each diagnosis and variant class, the top 30 KEGG pathways were defined as the core signature. Drugs were linked to diagnoses by pathway overlap, excluding on-label indications and infection-only supports, and ranked using a composite Repurposing Score integrating drug maturity/evidence, diagnosis-specific pathway concordance, and WGS-derived genetic support. Score-weight sensitivity analyses increased the contribution of genetic support. Evidence support was assessed by automated screening of publications and ClinicalTrials.gov records. Collectively, 906 drug-linked shared pathways yielded 12,040 unique repurposing pairs; 25.4% were supported by three or more variant classes. Downstream validation-priority annotation identified 1430 high-confidence drug–diagnosis pairs after stratifying candidates by cohort size, variant-class support, supporting-pathway count, and broad KEGG disease-module support. Frequently implicated mechanisms included RTK-MAPK/PI3K, VEGF, immune/checkpoint, and neurotrophin signaling. Variant-class-resolved WGS pathway prioritization coupled to curated pharmacology enables scalable cross-diagnosis repurposing using existing drugs, recovering clinically explored strategies and generating genetically supported hypotheses for biomarker-guided validation.

## Introduction

CNS-related conditions present a wide definition of medical conditions, spanning primary brain tumors, neurodevelopmental disorders, cerebrovascular disease, and major psychiatric syndromes. While progress has been made, the field remains disproportionately slow and failure-prone relative to other therapeutic areas. A central bottleneck is drug exposure, the blood-brain barrier (BBB), is a dynamic interface that restricts entry of many systemically administered agents through tight junctions, active efflux, and metabolic enzymes, and it can remodel in disease, further complicating delivery strategies [1,2]. Even when compounds reach the CNS, translation from preclinical models to patients is limited by incomplete disease models, pronounced biological heterogeneity, and uncertainty in dose-response relationships at clinically achievable CNS concentrations [3,4]. These constraints contribute to high attrition and leave many CNS-related conditions without disease-modifying options. Lack of new CNS therapies amplifies the urgency for alternative strategies.

Drug repurposing offers a practical route to expand the therapeutic repertoire under these constraints. By leveraging compounds with established manufacturing, safety, and human pharmacokinetic information, repurposing can shorten timelines and reduce development risk compared with de novo discovery. For CNS indications, BBB penetration and CNS exposure remain essential downstream triage criteria and are not assumed from approval status alone [5]. The strategy is most compelling when candidate drugs have evidence of CNS exposure or when they act on peripheral vascular or immune processes that secondarily shape CNS pathology. Importantly, BBB penetration cannot be assumed even for approved agents; however, existing CNS pharmacokinetic/pharmacodynamic measurements (e.g., cerebrospinal fluid or brain exposure where available) and dose-limiting toxicities can be used to triage feasibility and to design exposure-matched trials. Because BBB exposure data are incomplete at scale, the present framework prioritizes mechanism-level hypotheses and clinical maturity; BBB feasibility is treated as a downstream triage criterion in validation. Repurposing frameworks that integrate mechanism and clinical feasibility have therefore been advocated to reduce “trial-and-error” translation, particularly for heterogeneous neurological diseases [6,7].

Recent CNS-focused repurposing efforts illustrate feasibility and the need for systematic prioritization. In neuro-oncology, targeted therapies developed for systemic cancers have been evaluated in primary CNS tumors when actionable pathway drivers are present. For example, selective TRK inhibition with Larotrectinib shows clinical activity in TRK fusion-positive primary CNS tumors [8]. Immuno-oncology agents have also been tested in glioblastoma. The phase III CheckMate 143 trial benchmarked nivolumab against bevacizumab in recurrent disease, underscoring both clinical plausibility and the challenge of identifying responsive subgroups [9]. Beyond tumors, anti-angiogenic therapy has been repurposed for neurovascular pathology; a recent systematic review and meta-analysis support bevacizumab for radiation necrosis after brain radiotherapy, consistent with the role of VEGF signaling in vascular permeability and oedema [10]. These examples demonstrate that repurposing can succeed when biological rationale and exposure constraints are addressed, but candidate selection is unlikely to scale across the breadth of CNS-related conditions.

A major need is therefore for a systematic, mechanism-anchored view of repurposing opportunities that can integrate modern human genetics with actionable pharmacology while reserving BBB penetration and CNS exposure assessment for downstream validation. Whole-genome sequencing (WGS) increasingly indicates that risk for many neurodevelopmental and psychiatric phenotypes is enriched in noncoding regulatory elements and untranslated regions, alongside protein-altering and splice-disrupting variants [[11], [12], [13]]. These observations argue against repurposing strategies that rely solely on coding drivers or single-gene targets, and instead motivate pathway-level integration that can accommodate distributed, multi-class perturbation of shared signaling programs.

In this study, we construct a globally scoped, pathway-annotated catalogue of pharmacologic interventions relevant to CNS-related conditions and combine it with WGS-derived, diagnosis-specific KEGG pathway weights estimated across 92 CNS-related diagnoses. Although the 92 diagnoses are clinically heterogeneous and should not be interpreted as a single disease entity, this breadth is intentional, as the aim of the study is to test whether diagnosis-specific genomic perturbations may reveal recurring actionable pathways that could inform drug repurposing across clinically distinct disorders. By linking drugs to compact, mechanism-aligned KEGG signatures and intersecting these signatures with diagnosis-specific model-prioritized KEGG pathway sets inferred from patient genomes across exonic, splice-site, regulatory, and UTR variant classes, we generate a ranked space of repurposing hypotheses. The framework is designed to recover clinically explored strategies while also producing biologically grounded candidates for diagnoses with limited therapeutic options, enabling prioritized experimental validation and biomarker-informed trial design.

## Methods

**Fig. 1:**
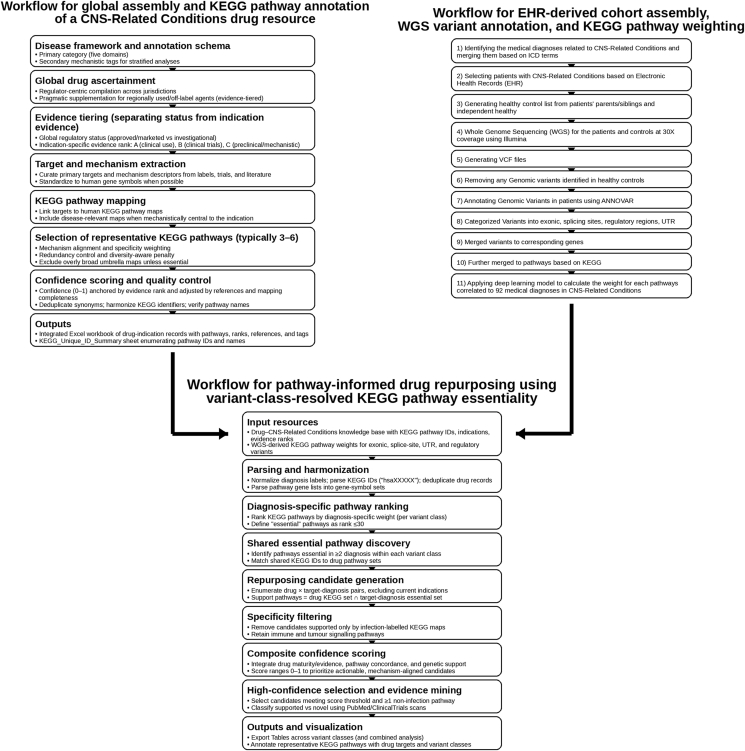
**Flow chart of the study design.** The workflow includes (A) curation of approved and clinical-stage drugs and mapping of drug targets to KEGG pathways; (B) inference of diagnosis-specific KEGG pathway weights from WGS across 92 diagnoses using a neural network model stratified by variant class; and (C) integration of (A) and (B) to prioritize repurposing candidates based on overlap between drug KEGG signatures and diagnosis-level model-prioritized KEGG pathway sets.

The flowchart of the study is illustrated in Fig. 1. Additional methodological details are provided in the Supplementary Methods.

### Workflow to extract previously developed and approved/clinical trial medications for CNS-related conditions on a global scale

#### Drug selection and KEGG annotation

A curated, globally ascertained catalogue of approved and clinical-stage therapeutics used for CNS-related conditions was created and annotated with mechanism-aligned KEGG pathway signatures (Supplementary Table 1). Pharmaceutically active medical compounds were identified through regulator-centric sources spanning major jurisdictions (e.g., Drugs@FDA, EMA/EPAR, MHRA, Health Canada, TGA, Swissmedic, PMDA and NMPA) and supplemented with regionally used or off-label agents when supported by documented clinical use and/or credible mechanistic evidence. Each drug-indication entry was annotated with global regulatory status, a maturity rank (A, marketed; B, clinical trials; C, other support), and an indication-specific evidence rank to separate overall development stage from evidence for the specific brain condition. Primary molecular targets and mechanism descriptors were standardized to human gene symbols where feasible and mapped to human KEGG pathways as a shared mechanistic vocabulary [14]. To enable interpretable pathway-overlap analyses, each drug-indication record was distilled to a compact mechanism signature consisting of representative KEGG pathway IDs chosen to maximize specificity and limit redundancy from ubiquitous signaling modules. Drug-pathway mapping was performed before integration with WGS-derived pathway profiles. For each drug-indication record, molecular targets and mechanism descriptors were extracted from regulatory documents, curated drug resources, clinical-trial records, and peer-reviewed studies. Targets were standardized to human gene symbols where possible and linked to human KEGG pathway maps. When a drug mechanism could not be represented by a single gene target, such as antibody-mediated cell depletion, ligand sequestration, receptor-complex modulation, or complex mixtures, mechanism-level descriptors were retained and mapped to KEGG pathways only when the pathway reflected the curated mechanism or disease-relevant biology. Representative KEGG pathways were selected based on mechanism alignment, pathway specificity, redundancy control, and exclusion or down-weighting of broad umbrella pathways unless they were central to the therapeutic mechanism.

### Workflow for identifying EHR-derived cohort & selection of genomic variants from WGS

To reduce redundancy introduced by ICD-10 granularity, CNS–related ICD codes were collapsed into phenotype-level groups after filtering for genetic or partially genetic relevance, removal of sex chromosome–linked disorders, and exclusion of non-brain diagnoses. Merging was performed using a rule-based phenotype normalization strategy that prioritized shared clinical meaning over anatomical or severity sub-coding. ICD rows that differed only by location, laterality, severity, remission state, intractability, or status modifiers were assigned to a common merged phenotype. Chromosomal deletion, duplication, and microdeletion syndromes were excluded before phenotype aggregation to avoid copy-number-variant–driven categories in this version of the analysis. The final phenotype-level table preserves the original ICD rows, their assigned merged phenotype, the merged ICD-code set, representative descriptions, the consensus primary CNS category, and the union of secondary tags, yielding 254 ICD rows collapsed into 92 merged phenotypes. Details of ICD preprocessing are provided in the Supplementary Methods.

Diagnosis-specific pathway profiles were derived from WGS of 4392 individuals across 92 CNS-related diagnoses, with the merged phenotype structure and cohort-size annotations summarized in Supplementary Table 2A and the ICD-level diagnostic terms listed in Supplementary Table 2B. A total of 1414 healthy controls was used as background. Case/control definitions, diagnosis assignment rules, and sequencing/batch covariates are described in Supplementary Methods; covariates were controlled to reduce confounding in pathway weight estimation. Controls were selected to minimize neurological phenotypes and to match sequencing platform/batch; residual differences were evaluated in sensitivity analyses. KEGG pathway weight matrices were analyzed using a neural network model in parallel across four genomic feature classes: exonic coding, splice-site, untranslated region (UTR), and noncoding regulatory variation, using ENCODE-linked candidate *cis*-regulatory element annotations to support interpretation of regulatory variants [11]. ENCODE-linked regulatory variants were analyzed as a separate variant class. Because the 92 CNS-related diagnoses span neuronal, glial, vascular, immune, and neoplastic biology, regulatory annotations were not restricted to a single neuronal or brain tissue source. Regulatory-variant findings were therefore interpreted as pathway-prioritization signals and evaluated together with variant-class support, mutation fraction, recurrently mutated gene count, and simulation-derived stability metrics. Future extensions using cell-type-resolved brain, glial, vascular, immune, and tumor regulatory maps may further refine regulatory variant-to-gene assignment. The neural network training objective, input feature construction, and evaluation scheme, including patient-level splits to prevent diagnosis leakage, are described in Supplementary Methods; all model selection was performed without access to drug annotations. Pathway feature attribution was based on repeated permutation importance rather than direct interpretation of hidden-layer weights. Permutation importance was repeated across 20 simulations, and mean importance and simulation-derived standard deviation were retained as stability measures. These pathway weights are interpreted as model-prioritized signals for candidate enumeration, not as proof of causal pathway dependence. For each pathway, matrices included simulation-derived stability statistics (mean and standard deviation across 20 simulations) alongside diagnosis-specific pathway weights from the neural network model. For each diagnosis and variant class, pathway-level features summarize aggregated variant burden or importance across genes in each KEGG pathway. Simulations reflect resampling and model re-fitting; performance metrics are reported per diagnosis and variant class. KEGG identifiers were normalized to support deterministic joins across resources. Variants were mapped to genes by functional class: exonic and splice-site variants to the affected transcript; UTR variants to the corresponding gene; regulatory variants to ENCODE-linked candidate *cis*-regulatory elements assigned to target genes. Gene sets were then aggregated to KEGG pathways using KEGG gene membership. Variant-to-pathway mapping was implemented as a deterministic post-annotation workflow. Variants passing quality-control and recurrence filters were first assigned to genes according to genomic context. Exonic, splice-site, and UTR variants were assigned to the affected transcript or corresponding gene, whereas regulatory variants were assigned using ENCODE candidate *cis*-regulatory element annotations and linked target-gene information where available. Gene identifiers were harmonized before pathway aggregation, including conversion of HGNC symbols or Ensembl identifiers to Entrez Gene IDs. Entrez identifiers were then joined to human KEGG pathway membership using a locally cached KEGG pathway-gene association table. For each KEGG pathway, duplicate genes were removed, and the pathway-level output retained the KEGG pathway ID, pathway name, recurrently mutated gene count, recurrently mutated gene list, mutation fraction, mean pathway importance, simulation-derived standard deviation, and diagnosis-specific pathway weight.

For each diagnosis within each variant class, KEGG pathways were ranked by the diagnosis-specific weight score; the top 30 pathways were defined as the core pathway signature for candidate enumeration. This threshold was selected to balance specificity and coverage across heterogeneous diagnoses and variant classes: a narrower top-10 set may miss distributed but druggable pathway perturbations, whereas a broader top-50 set may increase non-specific overlap with broad KEGG signaling or disease modules. The core pathway signature is used operationally and does not imply experimentally validated pathway essentiality, causal sufficiency, treatment response, or direct therapeutic dependence. We report pathway rank stability across simulations, including the fraction of runs in which a pathway appears in the top 30, to support robustness of the selected set. To support interpretation across heterogeneous diagnostic groups, pathway outputs were annotated with diagnosis-level cohort size and model-stability metrics. These annotations included pathway-weight magnitude, mean pathway importance, simulation-derived standard deviation, mutation fraction, recurrently mutated gene count, and supporting variant-class count. These fields were used to stratify pathway outputs for validation prioritization and to distinguish broadly reinforced signals from pathways driven by limited cohort or variant support.

### Workflow for repurposing drug candidates based on model-prioritized KEGG pathways

Candidate drug-diagnosis pairs were enumerated by intersecting each drug's KEGG signature with each target diagnosis' model-prioritized KEGG pathway set, excluding cases where the target diagnosis overlapped the drug's current indication to focus on non-redundant repurposing hypotheses. Overlap was defined using diagnosis-group mappings linking each drug's curated indications to the 92 diagnosis labels; pairs were excluded when the target diagnosis matched the drug's labelled or curated indication group. Because infectious-disease KEGG maps can act as noises for generic inflammatory signaling, candidates supported exclusively by infection-labelled pathways were removed while retaining immune and tumor signaling pathways. Meanwhile, to mitigate inflation and reduced interpretability introduced by KEGG organ-labelled cancer disease maps, which can act as surrogates for generic growth and survival circuitry, we applied an additional disease-module specificity filter after infection filtering. For non-neoplastic target diagnoses, candidates were removed when all non-infection supporting pathways were cancer disease maps; candidates for neoplastic target diagnoses retained cancer disease maps. Candidates were ranked using a composite Repurposing Score that integrated three scaled components: drug maturity/evidence (D), pathway concordance in the target diagnosis (P), and WGS-derived genetic support (M). The D component captured global drug maturity, indication-specific evidence rank, and curated confidence score. The P component captured the strength of overlap between the drug's KEGG signature and diagnosis-specific model-prioritized KEGG pathways. The M component captured WGS-derived support from pathway importance, recurrent-variant burden, mutation fraction, and variant-class support among the supporting pathways. The primary Repurposing Score was defined as 0.55·D + 0.30 P + 0.15 M. This weighting was selected to prioritize repurposing actionability among approved or clinical-stage drugs while retaining pathway concordance and WGS-derived support as required components of candidate ranking. Because component weights can influence candidate ordering, ranking sensitivity was evaluated using alternative weighting schemes. These included balanced, genetics-forward, genetics-dominant, and pathway/genetic-forward scores. For each scheme, candidates were re-ranked, and rank stability was summarized using rank shift, top-k overlap, and Spearman correlation relative to the primary ranking. Candidates that remained highly ranked under genetics-forward scoring were annotated as weight-stable, genetically supported repurposing hypotheses. The sensitivity analysis is reported in Supplementary Table 4D and summarized in Supplementary Fig. S1. Under genetics-forward weighting, only 6 of the primary top 100 candidates were retained, and the Spearman rank correlation with the primary ranking was 0.648. Increasing the contribution of WGS-derived genetic support did not simply reproduce the actionability-weighted ranking, but identified a complementary candidate space enriched for stronger genetic reinforcement.

Because the pipeline evaluates thousands of drug-diagnosis hypotheses, the score is presented as a prioritization heuristic rather than a formal significance test. Candidate drug–diagnosis pairs were further annotated with downstream support metrics to guide validation prioritization. These metrics included target-diagnosis cohort size, number of supporting KEGG pathways, number of non-infection supporting pathways, number of non-cancer/non-infection supporting pathways, number of supporting variant classes, mean supporting pathway weight, mean mutation fraction, and simulation-derived pathway variability. Candidate pairs were assigned validation-priority flags when support was limited to a small diagnostic cohort, a single pathway, a single variant class, high pathway-importance variability, or broad KEGG disease-module overlap. High-confidence candidates were defined by Repurposing Score ≥0.75, drug rank A/B, at least two supporting pathways, at least one non-infection pathway, and support from at least two variant classes, with additional separation of candidates supported only by broad metabolism modules, very-small-cohort labels, clinically sensitive diagnostic labels, or broad disease-module-only overlap. High-confidence candidates were further annotated according to whether their ranking remained stable under genetics-forward weighting. Collapsing retained a unique drug-diagnosis pair with the union of supporting pathways across classes and the per-class scores to avoid double-counting evidence. Evidence support was assessed by automated term-based screening of PubMed and ClinicalTrials.gov using diagnosis synonyms and drug names, followed by manual confirmation of matches for each high-confidence pair; matched PMIDs and/or NCT identifiers were retained for audit, consistent with best-practice recommendations for systematic repurposing workflows [5]. This screening was used for plausibility classification and does not constitute independent model validation.

## Results

Across exonic, regulatory, UTR, and splice-site variant classes, we identified 906 shared model-prioritized KEGG pathways that were simultaneously high-weighted across CNS-related diagnoses and linked to at least one approved or clinical-stage compound (Supplementary Table 3A). Diagnosis-level pathway annotations by variant class, cohort size, and attribution stability are provided in Supplementary Table 3B. When drug-pathway annotations were paired with diagnosis-specific model-prioritized pathway sets and the cancer disease-map filter for non-neoplastic targets, we obtained 22,722 row-level candidate entries across variant classes (Supplementary Table 4A) and 12,040 unique drug–diagnosis pairs after collapsing across variant classes (Supplementary Table 4B). Cohort-size sensitivity analyses for the collapsed candidate set are provided in Supplementary Table 4C. Because diagnosis-level cohort sizes varied across the 92 CNS-related conditions, the candidate space was stratified using downstream validation-priority annotations. Candidate interpretation was therefore not based on cohort size alone. Downstream candidate annotations incorporated cohort-size bin, variant-class support, supporting-pathway count, pathway specificity, broad KEGG disease-module status, and score-weight sensitivity so that large-cohort signals could be distinguished from candidates supported by convergent genetic and pathway evidence.

Candidate pairs supported across multiple variant classes and multiple non-infection pathways were considered more strongly reinforced, whereas pairs supported by smaller cohorts, single variant classes, single pathways, or broad KEGG disease modules were retained in the full output with validation-priority flags. After downstream annotation, 1430 of 12,040 unique drug–diagnosis pairs met the high-confidence criteria. Excluding diagnoses in the very-small available-count bin did not reduce this high-confidence subset, whereas excluding both small and very-small available-count diagnoses retained 1185 high-confidence pairs. High-confidence candidates were distributed across both large and non-large diagnostic groups, with 662 pairs from large available-count diagnoses and 768 pairs from non-large diagnoses. To evaluate whether candidate ordering was overly dependent on the actionability-weighted primary score, rankings were recalculated under alternative score weights that increased the contribution of WGS-derived genetic support. The sensitivity analysis compared the primary weighting scheme with balanced, genetics-forward, genetics-dominant, and pathway/genetic-forward schemes. Candidate ordering changed when greater weight was assigned to WGS-derived support: the genetics-forward score retained 6 of the primary top 100 candidates and had a Spearman rank correlation of 0.648 with the primary ranking. These results indicate that the primary score and genetics-forward score emphasize complementary candidate groups. The primary score prioritizes near-term repurposing actionability among existing approved or clinical-stage drugs, whereas genetics-forward scoring highlights candidates with stronger WGS-derived pathway support. Full rank shifts, top-k retention metrics, and weight-sensitivity status are provided in Supplementary Table 4D and summarized in Supplementary Fig. S1. Variant evidence remained multi-modal: the mean number of supporting variant contexts per unique pair was 1.84, with 25.4% of pairs supported by three or more variant classes and 4.8% supported by all four. Filtering was necessary because many infection-labelled KEGG maps embed ubiquitous innate immune pathways and interferon signaling that can spuriously connect unrelated diagnoses.

The shared-pathway landscape was dominated by three recurring classes. First, receptor tyrosine kinase (RTK) and downstream kinase cascades, including ErbB signaling (hsa04012), MAPK/ERK, PI3K-Akt, and VEGF signaling (hsa04370), were repeatedly high-weighted across tumor and non-tumor diagnoses, consistent with the dual role of these pathways in oncogenesis and neurovascular remodeling. Second, adaptive and innate immune pathways, including T-cell differentiation modules, cytokine signaling, and checkpoint-related networks, formed another high-weight cluster spanning inflammatory, vascular, and neoplastic phenotypes, in line with accumulating evidence that neuroinflammation is a shared pathological component across diverse CNS-related conditions. Third, neuronal growth and plasticity programs exemplified by neurotrophins signaling (hsa04722) appeared as shared high-weight pathways across neurodevelopmental and neoplastic categories, consistent with the repurposing potential of developmental signaling drugs when dysregulated neuronal differentiation and survival pathways are implicated. The most interpretable shared pathway classes were those supported by multiple variant classes, recurrently mutated genes within the pathway, and non-infection/non-cancer drug-linked pathways. RTK-MAPK/PI3K signaling, VEGF signaling, neurotrophins signaling, and selected immune-modulatory pathways met these criteria across multiple diagnoses. Pathway overlaps driven primarily by broad disease maps, isolated metabolism modules, or single variant classes were retained in the supplementary output with validation-priority annotations, preserving the full discovery space while allowing readers to focus on the most strongly reinforced signals.

Within the evidence-supported candidate set (Table 1), we observed two translational motifs: indication-expansion examples within established CNS or neuro-oncology contexts and cross-domain repurposing hypotheses supported by shared pathway biology. The first motif comprised targeted oncology agents originally developed for systemic malignancies that have been evaluated in primary CNS tumors based on shared pathway dependencies. Larotrectinib, a selective TRK inhibitor, ranked as the top supported candidate for malignant neoplasm of brain, with pathway alignment to neurotrophins signaling and multi-context genetic support; clinical datasets in TRK-fusion positive primary CNS tumors show measurable and durable responses to Larotrectinib [8], and pooled analyses support a tissue-agnostic approach in TRK fusion cancers [15]. In parallel, immune checkpoint modulation emerged as a supported motif for malignant neoplasm of brain. Nivolumab was prioritized in our pathway-based ranking, and the phase III CheckMate 143 trial provides direct clinical evidence for PD-1 blockade in recurrent glioblastoma, benchmarking outcomes against bevacizumab [9]. Although overall survival was comparable in the trial population, the presence of this candidate among the top supported hits indicates that shared immune and cancer pathway essentiality can recover clinically tested strategies.

**Table 1:** Evidence-supported repurposing, indication-expansion, and within-domain candidate examples in CNS-related conditions.

Drug	Candidate category	Repurposing CNS-related conditions	Approved indication	Repurposing score	Clinical trials and/or evidence	Notes	Downstream support annotation
Larotrectinib	Evidence-supported precision oncology indication expansion/clinical benchmark	Malignant neoplasm of brain	NTRK fusion + tumors incl. CNS/brain mets	0.923	Targeted therapy evidence in TRK fusion-positive primary CNS tumors	Pooled analyses of TRK fusion-positive primary CNS tumors across larotrectinib trials report durable intracranial responses.	1 variant class(es); 1 supporting pathway(s); cohort bin: Large (>=1000); conservative support: No
Lapatinib	Evidence-supported oncology-to-neuro-oncology indication expansion	Malignant neoplasm of brain	HER2+ breast cancer; systemic cancer with intracranial disease contexts	0.872	Clinical trials in intracranial malignancy	Evaluated in recurrent/refractory CNS tumors and HER2-positive breast cancer brain metastases.	4 variant class(es); 2 supporting pathway(s); cohort bin: Large (>=1000); conservative support: Yes
Erlotinib	Evidence-supported oncology-to-neuro-oncology indication expansion	Malignant neoplasm of brain	EGFR-mutant NSCLC	0.846	Multiple clinical trials in glioblastoma	Evaluated as monotherapy and in combinations; biomarker stratification may be required.	3 variant class(es); 3 supporting pathway(s); cohort bin: Large (>=1000); conservative support: Yes
Gefitinib	Evidence-supported oncology-to-neuro-oncology indication expansion	Malignant neoplasm of brain	EGFR-mutant NSCLC	0.845	Clinical trial (phase II)	Open-label phase II study evaluated gefitinib in recurrent glioblastoma.	2 variant class(es); 1 supporting pathway(s); cohort bin: Large (>=1000); conservative support: No
Osimertinib	Evidence-supported CNS-active oncology indication expansion	Malignant neoplasm of brain	EGFR-mutant NSCLC with brain metastases	0.805	PMID:32547705		3 variant class(es); 2 supporting pathway(s); cohort bin: Large (>=1000); conservative support: Yes
Nivolumab	Evidence-supported neuro-oncology immunotherapy benchmark	Malignant neoplasm of brain	Melanoma/NSCLC brain metastases	0.799	Phase III trial in recurrent glioblastoma	CheckMate 143 compared nivolumab versus bevacizumab in recurrent glioblastoma.	3 variant class(es); 1 supporting pathway(s); cohort bin: Large (>=1000); conservative support: No
Bevacizumab	Evidence-supported within-domain neuro-oncology/neurovascular indication expansion	Neurofibromatosis	Radiation necrosis; recurrent glioma	0.924	PMID:31254266; PMID:31626572		1 variant class(es); 3 supporting pathway(s); cohort bin: Large (>=1000); conservative support: No
Imatinib	Evidence-supported cross-domain vascular repurposing	Other cerebral infarction	Systemic cancer with intracranial disease contexts	0.844	Phase II randomized trial + registered phase III trial in acute ischemic stroke	Signals on neurological outcomes and a registered phase III placebo-controlled trial.	3 variant class(es); 5 supporting pathway(s); cohort bin: Very small (<50); conservative support: No
Natalizumab	Evidence-supported cross-domain CNS repurposing: Neuroimmunology to neurovascular disease	Cerebral infarction	Relapsing MS	0.869	Randomized, placebo-controlled trials in acute ischemic stroke	ACTION and ACTION II tested IV natalizumab within 24 h of AIS.	4 variant class(es); 3 supporting pathway(s); cohort bin: Moderate (200–999); conservative support: No
Minocycline	Evidence-supported cross-domain neuroinflammatory/neurovascular repurposing	Cerebral infarction	Stroke neuroprotection trials; neuroinflammation models	0.752	Clinical trials in acute ischemic stroke; MINOS; early-phase ICH trial	Multiple clinical studies have assessed minocycline in acute ischemic stroke.	2 variant class(es); 2 supporting pathway(s); cohort bin: Moderate (200–999); conservative support: Yes

Candidate categories distinguish evidence-supported indication expansion, within-domain CNS/neuro-oncology repurposing, clinical benchmark examples, and cross-domain repurposing hypotheses. These labels clarify the translational context of each candidate and do not change the underlying pathway-prioritization results. Indication-expansion and clinical benchmark examples remain important because they show that the framework recovers clinically plausible and previously tested strategies, whereas cross-domain examples highlight broader repurposing opportunities across distinct CNS-related disease areas.

The second evidence-supported motif involved immunomodulatory agents prioritized for cerebrovascular phenotypes, consistent with core pathways capturing leukocyte trafficking and endothelial dysfunction. Natalizumab was prioritized for cerebral infarction, aligning with immune cell migration and inflammatory signaling pathways that are essential in subsets of cerebrovascular diagnoses. In addition, imatinib was repeatedly prioritized for cerebral infarction and related occlusion/stenosis phenotypes, consistent with the broader concept that modulation of vascular and inflammatory signaling may be beneficial in acute and subacute cerebrovascular injury, aligning with reported clinical investigation in acute ischemic stroke (Table 1). While several of these agents have heterogeneous trial outcomes and safety constraints, their emergence from an unbiased pathway-overlap screen indicates that the WGS-derived pathway weights capture clinically relevant biology beyond classical tumor contexts.

**Fig. 2:**
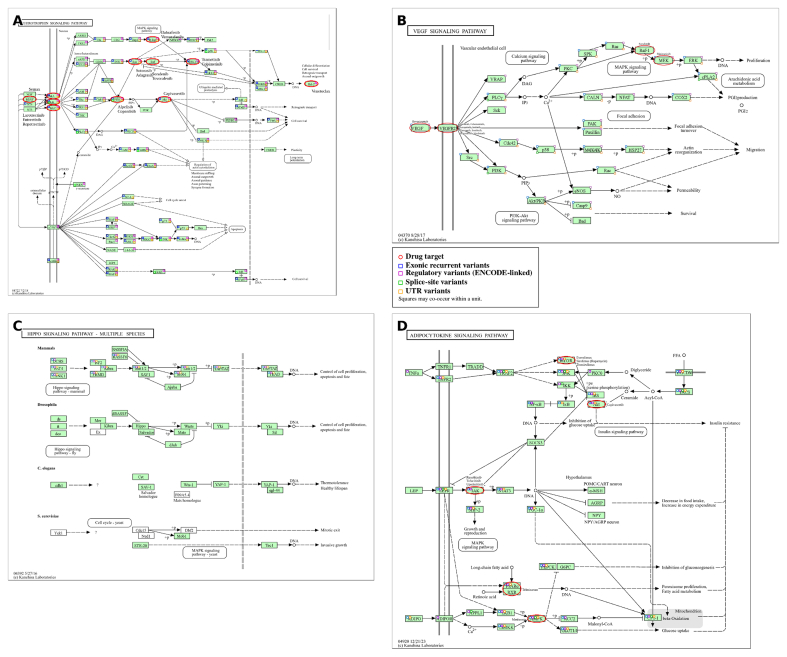
**Variant genetic support and drug-target overlay on representative KEGG pathways prioritized for repurposing across CNS-related conditions.** (A) **Neurotrophin signaling** (hsa04722), highlighting TRK receptors (NTRK1/2/3) and downstream MAPK and PI3K-AKT nodes as tractable intervention points. (B) **VEGF signaling** (hsa04370), emphasizing VEGFA-VEGFR2 signaling and the RAF-MEK cascade implicated in endothelial activation, permeability, and vascular remodeling. (C) **Hippo signaling** (hsa04392), depicting the conserved MST/LATS-YAP/TAZ-TEAD growth-control module across species; no direct drug-target node is shown in the KEGG schematic for Hippo-linked agents. (D) **Adipocytokine signaling** (hsa04920), illustrating immunometabolic crosstalk through JAK/STAT, PI3K-AKT-mTOR, AMPK, and PPARα/RXR hubs. Red circles denote pathway nodes targeted by drugs in the curated compendium; colored squares indicate genes harboring variants in the WGS cohort, stratified as exonic (blue), regulatory/ENCODE-linked (purple), splice-site (green), and UTR (orange). Multiple variant classes may co-occur at a single node. Pathway maps are adapted from KEGG (Kanehisa Laboratories) with study-specific overlays.

To illustrate how genetic evidence distributes across pathways, Fig. 2 depicts four representative, evidence-supported examples spanning RTK signaling, immune checkpoint signaling, neuronal growth factor signaling, and angiogenic signaling. In each panel, drug-targeted nodes (red circles) coincide with pathway entry or control points, while variants appear across upstream and downstream members across variant classes. For example, ErbB signaling shows exonic and regulatory/UTR variant support across adaptor proteins and downstream kinases, indicating that genetic support spans multiple nodes and variant classes within the pathway. A similar distributed pattern is observed for checkpoint and neurotrophins signaling, consistent with models in which regulatory and splice-site variants modulate immune and neuronal pathway tone rather than producing single dominant coding lesions. Finally, VEGF signaling shows multi-class variant support across kinase and phospholipase mediators, complementing evidence that anti-VEGF therapy can reduce neurovascular permeability and oedema in brain disease contexts [10]. Selected high-priority repurposing hypotheses without direct prior indication evidence are shown in Table 2, with the full collapsed candidate set and validation-priority flags provided in Supplementary Table 4B. Rank stability under genetics-forward weighting is provided in Supplementary Table 4D. Together, the results indicate that pathway-sharing across diagnoses, reinforced by coding and noncoding variation, can yield a ranked repurposing space that recovers clinically explored strategies and nominates mechanistically grounded hypotheses for validation.

**Table 2:** Selected conservative repurposing hypotheses without direct prior indication evidence.

Drug	Repurposing CNS-related conditions	Approved/current indication	Repurposing score	Genomic variant types	KEGG pathways	Downstream support annotation	Weight sensitivity	D score	P score	M score
Neratinib	Microcephaly	HER2+ breast cancer	0.925	Exonic; Splice_site; UTR	Glucagon signaling pathway - Homo sapiens (human); GnRH signaling pathway - Homo sapiens (human)	3 variant class(es); 2 supporting pathway(s); 2 non-broad pathway(s); cohort bin: Large (>=1000)	Primary-score enriched	0.930	1.000	0.129
Binimetinib	Neurofibromatosis	BRAF V600 melanoma (combo); Systemic cancer with intracranial disease (brain metastases/leptomeningeal) contexts; CNS activity varies	0.923	Splice_site; UTR	VEGF signaling pathway - Homo sapiens (human); Apoptosis - Homo sapiens (human)	2 variant class(es); 2 supporting pathway(s); 2 non-broad pathway(s); cohort bin: Large (>=1000)	Primary-score enriched	0.930	1.000	0.100
Encorafenib	Cerebral vascular malformations	BRAF V600 melanoma; Systemic cancer with intracranial disease (brain metastases/leptomeningeal) contexts; CNS activity varies	0.922	Exonic; Splice_site; UTR	IL-17 signaling pathway - Homo sapiens (human); TNF signaling pathway - Homo sapiens (human)	3 variant class(es); 2 supporting pathway(s); 2 non-broad pathway(s); cohort bin: Small (50–199)	Primary-score enriched	0.930	1.000	0.076
Sodium oligomannate	Personality/behavior disorders due to brain disease	Alzheimer's disease (China-approved; global development history)	0.921	Splice_site; UTR	Toll-like receptor signaling pathway - Homo sapiens (human); NF-kappa B signaling pathway - Homo sapiens (human)	2 variant class(es); 2 supporting pathway(s); 2 non-broad pathway(s); cohort bin: Moderate (200–999)	Primary-score enriched	0.930	1.000	0.055
Binimetinib	Arnold-Chiari malformation	BRAF V600 melanoma (combo); Systemic cancer with intracranial disease (brain metastases/leptomeningeal) contexts; CNS activity varies	0.913	Exonic; Splice_site; UTR	HIF-1 signaling pathway - Homo sapiens (human); VEGF signaling pathway - Homo sapiens (human)	3 variant class(es); 2 supporting pathway(s); 2 non-broad pathway(s); cohort bin: Moderate (200–999)	Primary-score enriched	0.930	1.000	0.100
Repotrectinib	Somatoform disorders	ROS1+ NSCLC (CNS activity); Systemic cancer with intracranial disease (brain metastases/leptomeningeal) contexts; CNS activity varies	0.912	Exonic; Regulatory; Splice_site; UTR	Retinol metabolism - Homo sapiens (human); Terpenoid backbone biosynthesis - Homo sapiens (human); Nitrogen metabolism - Homo sapiens (human); Sulfur metabolism - Homo sapiens (human); Porphyrin metabolism - Homo sapiens (human); Proteasome - Homo sapiens (human)	4 variant class(es); 6 supporting pathway(s); 1 non-broad pathway(s); cohort bin: Moderate (200–999)	Primary-score enriched	1.000	0.999	0.417
Binimetinib	Other congenital malformations of brain	BRAF V600 melanoma (combo); Systemic cancer with intracranial disease (brain metastases/leptomeningeal) contexts; CNS activity varies	0.911	Exonic; Splice_site	HIF-1 signaling pathway - Homo sapiens (human); VEGF signaling pathway - Homo sapiens (human)	2 variant class(es); 2 supporting pathway(s); 2 non-broad pathway(s); cohort bin: Large (>=1000)	Primary-score enriched	0.930	1.000	0.100
Lapatinib	Other cerebrovascular disease	HER2+ breast cancer (CNS use); Systemic cancer with intracranial disease (brain metastases/leptomeningeal) contexts; CNS activity varies	0.910	Exonic; Splice_site	Fc epsilon RI signaling pathway - Homo sapiens (human); C-type lectin receptor signaling pathway - Homo sapiens (human)	2 variant class(es); 2 supporting pathway(s); 2 non-broad pathway(s); cohort bin: Moderate (200–999)	Primary-score enriched	0.930	1.000	0.086
Lorazepam (IV)	Personality/behavior disorders due to brain disease	Status epilepticus	0.909	Exonic; UTR	Primary bile acid biosynthesis - Homo sapiens (human); Steroid hormone biosynthesis - Homo sapiens (human); JAK-STAT signaling pathway - Homo sapiens (human)	2 variant class(es); 3 supporting pathway(s); 1 non-broad pathway(s); cohort bin: Moderate (200–999)	Stable top-100 under primary and genetics-forward scoring	1.000	0.911	0.569
Lorazepam (IV)	Bipolar disorder	Status epilepticus	0.909	Exonic; Regulatory; UTR	Fatty acid degradation - Homo sapiens (human); Ubiquinone and other terpenoid-quinone biosynthesis - Homo sapiens (human); JAK-STAT signaling pathway - Homo sapiens (human); Steroid hormone biosynthesis - Homo sapiens (human)	3 variant class(es); 4 supporting pathway(s); 1 non-broad pathway(s); cohort bin: Moderate (200–999)	Stable top-100 under primary and genetics-forward scoring	1.000	0.871	0.649

Selected candidates met downstream high-confidence criteria, including Repurposing Score ≥0.75, drug rank A/B, at least two supporting pathways, at least one non-infection supporting pathway, support from at least two variant classes, and no metabolism-only, very-small-cohort, clinically sensitive-label, or broad disease-module-only support. Weight-sensitivity status indicates whether the candidate remained prioritized when WGS-derived genetic support received greater weight. “Without direct prior indication evidence” refers to the absence of direct drug–diagnosis evidence in targeted literature and ClinicalTrials.gov screening; it does not indicate discovery of a new drug and does not prove that no relevant evidence exists.

## Discussion

Drug repurposing is increasingly pursued as a practical route to expand therapeutic options while leveraging de-risked compounds, but it remains limited by the challenge of matching drugs to the heterogeneous biology of CNS-related conditions [5]. We provide a pathway-level framework that systematizes repurposing across CNS-related conditions by integrating curated drug-pathway annotations with diagnosis-specific KEGG pathway weights inferred from WGS cohorts. The design shifts the repurposing approach from single targets to shared pathway dependencies, providing a mechanistic substrate that is better aligned with polygenic and heterogeneous disease architectures [16].

A key implication of the results is that high-weighted pathways inferred from patient genomes can reveal partial pathway-level convergence among diagnoses that are clinically distinct. For example, RTK signaling, angiogenic programs, and immune signaling appeared repeatedly among shared model-prioritized pathways, consistent with pleiotropic roles of these processes in tumor growth, blood-brain barrier integrity, vascular remodeling, and neuroinflammation [17]. Importantly, our analyses were stratified by variant class (exonic, splice-site, regulatory, UTR) before integration, enabling the repurposing space to reflect both protein-altering lesions and noncoding mechanisms that tune expression and splicing. This is particularly relevant in brain phenotypes, where risk is frequently enriched in regulatory elements and untranslated regions rather than in coding sequences alone. Large-scale functional annotations such as ENCODE phase III provide the necessary regulatory maps to interpret these variants, and reviews of de novo noncoding regulatory mutations in neurodevelopmental disorders highlight the need to incorporate these classes into disease models [12]. By embedding regulatory and splice-site evidence alongside exonic variation, the pipeline better mirrors the true spectrum of WGS variation that may perturb pathway activity.

From a translational perspective, the study yields complementary outputs. Supplementary Table 3A serves as a cross-diagnosis atlas of shared drug-linked, model-prioritized KEGG pathways, while Supplementary Table 3B provides diagnosis-level pathway annotations by variant class, cohort size, and attribution stability. Supplementary Table 4A provides the row-level drug–diagnosis candidate output by variant class, Supplementary Table 4B provides the collapsed unique drug–diagnosis candidate set with downstream validation-priority annotations, Supplementary Table 4C summarizes cohort-size sensitivity analyses, and Supplementary Table 4D reports score-weight sensitivity analyses. Together, these outputs preserve the full repurposing hypothesis space while allowing investigators to prioritize candidates according to translational readiness, mechanistic specificity, WGS-derived support, and robustness to score weighting. This structure enables investigators to prioritize candidates based on the trade-off between readiness (approved vs clinical-stage), mechanistic specificity, and genetic reinforcement. The separation of these elements also facilitates sensitivity analyses, for example by increasing the weight assigned to genetic support in settings where multiple approved drugs exist, or conversely prioritizing maturity in settings where rapid clinical translation is the primary objective.

The evidence-supported top hits demonstrate that the framework can recover strategies already tested in modern CNS oncology and thereby provides a form of internal validation. For checkpoint inhibition, nivolumab was prioritized for malignant neoplasm of brain and the CheckMate 143 randomized trial offers direct evidence for PD-1 blockade in recurrent glioblastoma [9]. While the trial did not meet its primary survival endpoint, its conduct confirms that pathway-based prioritization can identify clinically plausible interventions. The framework also highlights why outcomes may vary. In this regard, Fig. 2 shows that variants supporting immune checkpoint pathway prioritization are distributed across multiple nodes and variant classes, implying that pathway-level activity may be modulated by diverse mechanisms and may depend on tumor microenvironmental context, antigenicity, and treatment-related immunosuppression. Similarly, TRK inhibition with larotrectinib illustrates a scenario where pathway prioritization aligns with a clearer genetic driver; clinical studies have reported activity in TRK fusion-positive primary CNS tumors, and pooled analyses support durable responses across fusion-positive tumors. In this setting, pathway alignment coupled to a well-defined driver alteration can yield a stronger therapeutic signal.

Beyond oncology, anti-angiogenic and vascular-targeted strategies emerged as evidence-supported indication-expansion or within-domain repurposing examples. Bevacizumab was prioritized through VEGF signaling overlap, and recent meta-analytic evidence supports its use for radiation necrosis after radiotherapy of brain metastatic disease, where reductions in oedema and clinical improvement are commonly observed. We therefore classify bevacizumab-related examples as evidence-supported indication-expansion or within-domain neuro-oncology/neurovascular repurposing rather than as novel cross-disciplinary repurposing hypotheses. Although radiation necrosis is not identical to the diagnoses represented in our WGS cohort, the pathway-level signal supports the broader concept that perturbations of neurovascular permeability and inflammatory remodeling can be pharmacologically modulated. The distributed variant support across VEGF pathway members in Fig. 2 further suggests that neurovascular phenotypes may arise from heterogeneous genetic perturbations converging on a shared angiogenic program, an architecture well-suited to pathway-level repurposing. The score-sensitivity analysis provides a practical framework for interpreting representative high-ranking candidates. Evidence-supported oncology examples such as larotrectinib and bevacizumab serve as internal benchmarking cases because their pathway assignments correspond to established CNS-relevant mechanisms: neurotrophin/TRK signaling in genetically defined CNS tumors and VEGF signaling in neurovascular permeability and oedema biology. Genetics-forward scoring is particularly useful for identifying candidates whose ranking is not solely explained by drug maturity. Candidates supported by multiple variant classes and non-broad pathway overlap were prioritized for experimental follow-up even when the current drug indication originated outside neurology. These examples should be interpreted as pathway-matched repurposing hypotheses requiring validation in disease-relevant cellular, organoid, or patient-derived models rather than as immediate therapeutic recommendations.

Complementing the evidence-supported examples above, we highlight four high-confidence pathways that together illustrate why a pathway-informed, variant-class-resolved repurposing strategy is well suited to CNS-related conditions. These include Neurotrophins signaling (hsa04722), VEGF signaling (hsa04370), Hippo signaling (hsa04392), and Adipocytokine signaling (hsa04920). These pathways remained prioritized after applying the cancer disease-map-only filter, indicating that the representative mechanisms are not driven by organ-labelled cancer maps. These pathways span neuronal plasticity, neurovascular remodeling, growth-control programs, and metabolic-immune crosstalk, and they were prioritized because the same study design steps converged on them: diagnosis-specific KEGG pathway weights derived from WGS cohorts prioritized shared pathway signals across clinically distinct phenotypes; curated drug-pathway annotations nominated tractable intervention points; and stratified evidence from exonic, splice-site, regulatory, and UTR variation provided orthogonal reinforcement for pathway perturbation. Two pathways provide “known support” anchors that are already under contemporary clinical evaluation in CNS contexts, thereby benchmarking biological specificity. Neurotrophin signaling links directly to TRK receptors (NTRK1/2/3), where selective TRK inhibition has shown clinical activity in TRK fusion-positive primary CNS tumors and pooled phase 1/2 datasets support activity across tumor types. This is a clear example of the framework working as intended, where a neuronal growth factor axis is prioritized, clinically mature inhibitors map to a defined node, and the resulting hypothesis aligns with a genetically defined responder population. VEGF signaling provides a complementary “network phenotype” example, in which neurovascular permeability and inflammatory remodeling rather than a single coding driver may dominate. Bevacizumab-mediated VEGF-A neutralization has demonstrated clinical activity in radiation necrosis after brain radiotherapy, and randomized-trial syntheses in recurrent glioblastoma indicate consistent effects on radiographic response and progression endpoints despite variable survival impact [18]. In our pathway-level outputs, VEGF-pathway reinforcement typically reflects variants distributed across multiple upstream and downstream components and across coding and noncoding classes, supporting the rationale for pathway-level targeting in genetically heterogeneous neurovascular phenotypes. In contrast, Hippo and Adipocytokine signaling represent high-confidence, comparatively under-explored repurposing spaces that extend beyond the current “usual suspects” of RTK and angiogenesis and are therefore positioned as trial-generating hypotheses. Hippo signaling controls growth through MST/LATS kinases and YAP/TAZ-TEAD transcriptional programs; dysregulation is increasingly implicated in glioma biology [19], yet systematic evaluation of Hippo-directed intervention strategies in CNS-related conditions remains limited. Importantly, the pathway is now pharmacologically tractable, wherein first-in-human TEAD inhibitors have entered early-phase clinical evaluation in solid tumors [20], creating a realistic entry point to test whether genetically reinforced Hippo pathway prioritization can be evaluated in CNS disease, provided blood-brain barrier exposure and neurotoxicity constraints can be met. Adipocytokine signaling captures a second, conceptually distinct opportunity in which leptin/adiponectin, JAK-STAT, AMPK, and inflammatory nodes implicate the metabolic-immune interface as a shared vulnerability across neuropsychiatric and neurodegenerative phenotypes. Experimental work links impaired adiponectin receptor signaling to memory dysfunction and Alzheimer's disease-like pathologies [21], and meta-analytic evidence indicates altered adiponectin levels in major depressive disorder [22], supporting biological plausibility for pathway-centric intervention even when coding drivers are absent.

The selected candidates in Table 2 represent high-priority repurposing hypotheses without direct prior indication evidence in targeted keyword scans. Their value lies in nominating new drug–diagnosis pairings from existing approved or clinical-stage pharmacology, supported by pathway overlap, WGS-derived genetic evidence, and downstream validation-priority annotation. These hypotheses should be interpreted as prioritized starting points for experimental triage rather than immediate clinical recommendations. Their ranking reflects both the primary actionability-weighted score and sensitivity analyses that increase the contribution of WGS-derived genetic support. Candidates whose rank was more dependent on drug maturity remain available in the full supplementary output, whereas Table 2 highlights candidates with stronger combined support for follow-up validation. In particular, candidates supported by three or more variant classes may be attractive because multi-modal genetic reinforcement can indicate robust pathway perturbation. Multi-class support is treated as a prioritization feature; it may reflect convergent biology but can also reflect pathway size or annotation density, which is why pathway-support and score-sensitivity annotations are reported in the supplementary tables. The figure-based analysis reinforces an important mechanistic principle: drug targets tend to be located at pathway “control points,” while disease-associated variants distribute across the pathway [23]. This architecture creates two opportunities. First, upstream inhibition (e.g., receptor-level blockade) can dampen downstream activation despite heterogeneity in distal variants, motivating repurposing even when the causal variant is not directly targetable. Second, distributed variant patterns suggest rational combinations. Combination design is a hypothesis derived from pathway topology and is not explicitly optimized in the present scoring framework. For example, if variants cluster in parallel downstream branches, combining an upstream inhibitor with a branch-specific modulator may increase pathway control. This logic is consistent with the broader repurposing literature, where pathway-level models are often used to justify combination strategies and to explain differential sensitivity. In brain diseases, where blood-brain barrier penetration and neurotoxicity constrain dosing, pathway-guided combination design may be especially valuable to test efficacy at tolerable exposures.

Variant class-specific signals may map to different intervention strategies. Coding variants may justify targeting altered proteins directly, whereas regulatory/UTR variants may motivate interventions that modulate expression or pathway tone, potentially through epigenetic or transcriptional regulators. Splice-site variants add another layer: splicing dysregulation is increasingly implicated in neurodegeneration and neurological disease mechanisms [24], and population-scale analyses show that genetic variation can broadly reshape splicing landscapes. Where splice-site support is prominent in a pathway, therapies that influence RNA processing or that target isoform-specific vulnerabilities may be particularly relevant. Similarly, studies of noncoding mutation impact in autism highlight that both transcriptional and post-transcriptional regulatory disruptions can contribute to brain phenotypes [13], supporting the inclusion of regulatory and UTR variant classes as legitimate mechanistic evidence for pathway perturbation. Future extensions could incorporate cell-type-resolved pathway activity from single-cell transcriptomics, blood-brain barrier penetration metrics, and pharmacogenomic constraints to match pathway hypotheses to drug exposure and patient subgroups.

Several limitations should be considered in interpreting these results. First, the framework depends on existing pathway and regulatory annotations. KEGG pathway boundaries are useful as a shared mechanistic vocabulary, but they are imperfect proxies for cell-type-specific signaling, and many genes participate in different complexes depending on cellular context. This is particularly relevant for immune, inflammatory, metabolic, and cancer-associated KEGG modules, where broad pathway definitions can create non-specific overlaps. Infection-only filtering, cancer disease-map filtering for non-neoplastic targets, and downstream validation-priority annotations reduce but do not eliminate this limitation. Second, the 92 CNS-related diagnoses are intentionally broad and clinically heterogeneous. This breadth supports cross-diagnosis discovery of recurring actionable pathway programs, but the results should be interpreted at the diagnosis and pathway level rather than as a single unified CNS disease mechanism. Third, pathway-level overlap between a drug signature and a diagnosis-specific pathway set does not establish treatment response or clinical benefit. The Repurposing Score is a prioritization metric that integrates drug maturity, pathway concordance, and WGS-derived support; it is not a probability of efficacy. Fourth, regulatory variant interpretation is limited by the tissue and cell-type specificity of enhancer and promoter activity. ENCODE-linked cCRE annotations provide a useful regulatory framework, but the current implementation does not fully resolve cell-type-specific activity across neuronal, glial, vascular, immune, and tumor states and may introduce non-CNS regulatory links. Fifth, BBB penetration, CNS exposure, and neurotoxicity were not modeled directly and must be assessed during downstream pharmacologic triage. Finally, the drug-pathway annotations are curated and vary in granularity, with some drugs mapping cleanly to target genes and others exerting pleiotropic effects that are only partially represented by KEGG identifiers. The most important next steps are independent WGS replication, transcriptomic or proteomic confirmation of pathway activity, cell-type-resolved regulatory refinement, functional testing in pathway-matched cellular, organoid, or patient-derived models, CNS exposure and safety assessment, and biomarker-stratified clinical evaluation for the strongest candidates.

## Conclusions

In summary, we present an integrative framework that combines curated pharmacological knowledge with whole-genome sequencing-derived pathway perturbations across both coding and noncoding variants to systematically generate a prioritized hypothesis space for drug repurposing in CNS disorders. By constructing a cross-diagnostic pathway atlas, alongside a comprehensive set of candidate therapeutics and stratified high-priority examples, with and without prior external validation, we establish a structured and scalable foundation for systematic repurposing efforts and experimental prioritization. Importantly, the convergence of shared biological pathways, genetic support, and varying levels of clinical maturity provides a pragmatic and translationally relevant route to identify and evaluate therapeutic opportunities within biomarker-defined patient subgroups. At the same time, our findings underscore key challenges, including the need for cell-type–specific and context-dependent validation, rigorous safety assessment, and prospective clinical studies guided by pathway-informed biomarkers.

Taken together, this approach advances a genetics-informed strategy for therapeutic repurposing, bridging large-scale genomic data with actionable pharmacologic knowledge to accelerate precision medicine in CNS disease.

## Ethics approval and consent to participate

We confirm that all methods were carried out in accordance with relevant regulatory guidelines and regulations. All experimental protocols were approved by the Institutional Review Board (IRB) of the Children's Hospital of Philadelphia (CHOP). Informed consent was obtained from all subjects. If subjects are under 18, consent was obtained from a parent and/or legal guardian with assent from the child if 7 years or older.

## Consent for publication

Not applicable.

## Availability of data and materials

The WGS has been uploaded to database of Genotypes and Phenotypes (dbGaP, https://www.ncbi.nlm.nih.gov/gap/) with the accession number phs001661.v2.p1. The rest WGS data could be accessed at the Kids First Data Resource Portal (DRC, https://portal.kidsfirstdrc.org/login). The EHR data is available upon request due to patient privacy.

## Author contributions

Conceptualization and supervision, Y.L. and H.H; literature search, Y.L.; data preparation & analysis, Y.L., H.Q.Q., F.D.M., J.J.C., C.X., J.K., H.Q., K.O., K.N., J.G.; data interpretation, Y.L.; original draft writing, Y.L., H.Q.Q.; review and revision led by Y.L., and H.H, with all authors contributing and approving the manuscript.

## Funding

The study was supported by the Institutional Development Funds from the Children’s Hospital of Philadelphia to the Center for Applied Genomics, and The Children's Hospital of Philadelphia Endowed Chair in Genomic Research to HH.

## Declaration of competing interest

The authors declared no potential conflicts of interest with respect to the research, authorship, and/or publication of this article.

## Figures and Tables

**Figs1:**